# Asymmetric Intrastromal Corneal Ring Segments with Progressive Base Width and Thickness for Keratoconus: Evaluation of Efficacy and Analysis of Epithelial Remodeling

**DOI:** 10.3390/jcm12041673

**Published:** 2023-02-20

**Authors:** Abdelmajid Benlarbi, Sofiene Kallel, Clementine David, Raphael Barugel, Quentin Hays, Isabelle Goemaere, Roxane Cuyaubere, Marie Borderie, Vincent Borderie, Nacim Bouheraoua

**Affiliations:** 1 CHNO des Quinze-Vingts, IHU ForeSight, INSERM-DGOS CIC 1423, 28 Rue de Charenton, F-75012 Paris, France; 2 Sorbonne Université, INSERM, CNRS, Institut de la Vision, 17 Rue Moreau, F-75012 Paris, France

**Keywords:** cornea, keratoconus, intrastromal corneal ring segment, asymmetric, aberrometry, topography, epithelium

## Abstract

**Purpose:** The aim of this study is to describe visual outcomes and epithelial remodeling following the implantation of asymmetric intracorneal ring segments (ICRSs) of variable thickness and base width for the management of duck-type keratoconus. **Methods:** A prospective observational study of patients with duck-type keratoconus was conducted. All patients received one ICRS AJL PRO + implant (AJL Ophthalmic). We analyzed demographic and clinical data, anterior segment optical coherence tomography (AS-OCT) data and Scheimpflug camera images obtained with a Placido disc MS-39 (CSO, Firenze, Italy) one and six months after surgery to determine keratometric and aberrometric outcomes and epithelial remodeling. **Results:** We studied 33 keratoconic eyes. ICRS implantation significantly improved both corrected distance visual acuity (CDVA) and uncorrected distance visual acuity at six months, as assessed with the logMAR (minimum angle of resolution) system, from 0.32 ± 0.19 to 0.12 ± 0.12 (*p* < 0.001) and from 0.75 ± 0.38 to 0.37 ± 0.24 (*p* < 0.001), respectively. Overall, 87% of implanted eyes gained ≥ 1 line of CDVA, and 3% of patients (*n* = 1) lost one line of CDVA; 55% of eyes attained a manifest refraction spherical equivalent between +1.50 and −1.50 D. Epithelial remodeling was greater at the wider and thicker end (+11.33 µm ± 12.95; *p* < 0.001 relative to the initial value) than at the narrower and thinner end (+2.24 µm ± 5.67; *p* = 0.01). Coma aberration was significantly reduced from 1.62 ± 0.81 µm to 0.99 ± 0.59 µm (*p* < 0.001). **Conclusions:** AJL-PRO + ICRS implantation for duck-type keratoconus improves refractive, topographic, aberrometric and visual parameters and induces progressive epithelial thickening along the segment.

## 1. Introduction

Keratoconus is a bilateral asymmetric ectatic disorder of the cornea characterized by localized corneal thinning and steepening, inducing irregular astigmatism that leads to visual impairment [1,2]. Intracorneal ring segment (ICRS) implantation is currently used in keratoconus treatment strategies; the efficacy and safety of this approach have been widely demonstrated [3,4,5,6,7]. Different types of ICRSs are available for different types of keratoconus. ICRSs may be symmetric or asymmetric, with different arc lengths, diameters, cross-sectional shapes, thicknesses and widths. Symmetric ICRS implantation has yielded good refractive and topographic astigmatism correction in patients with asymmetric keratoconus but does not optimally manage coma-like aberration in these patients, potentially limiting visual improvement [8,9,10,11]. Different types of ICRSs have recently been developed for the treatment of asymmetric keratoconus. The thickness or width of these segments varies so as to treat the keratoconus heterogeneously, targeting certain areas of the cornea, according to the phenotype. Asymmetric ICRS implantation has resulted in good visual, refractive and topographic outcomes, with a particularly large decrease in coma-like aberration [6,9,12,13,14,15].

Many studies have shown that ICRS implantation can be performed in combination with corneal cross-linking (CXL) or topography-guided or conventional photorefractive keratectomy (TG-PRK or PRK) to optimize visual rehabilitation [16,17,18]. Transepithelial laser procedures with a uniform epithelial ablation of 50 μm are the most widely used, as this approach is also used in protocols without ICRS implantation (Cretan Protocol or Athens Protocol) [19,20,21]. However, few studies have investigated epithelial remodeling after ICRS implantation, and there are currently no published data concerning epithelial remodeling after asymmetric ICRS implantation. Reinstein et al. showed that ICRS implantation for myopia treatment induced epithelial filling in the concave anterior stromal groove [22]. In anterior-segment optical coherence topography (AS-OCT) analyses on different types of keratoconus, David et al. showed that epithelial thickening in the internal zones contiguous to a symmetric ICRS differed between ICRS types, and reported epithelial thinning above the ICRS, with no smoothing of this effect after six months of follow-up [23]. Analyses of epithelium remodeling after asymmetric ICRS implantation are useful for determining whether the cornea has been regularized by ICRS implantation and for analyses of the asymmetric stromal effect of the asymmetric ICRS.

Here, we analyzed epithelial remodeling after AJL PRO + asymmetric ICRS (AJL Ophthalmic, Vitoria-Gasteiz, Spain) implantation by AS-OCT and with a Scheimpflug camera equipped with a Placido disc MS-39 (CSO, Firenze, Italy). The treated patients had duck-type keratoconus, defined as a paracentral topographic keratoconus phenotype with noncoincident topographic and coma axes. The visual, refractive, keratometric and aberrometric outcomes were also analyzed.

## 2. Patients and Methods

### 2.1. Study Design

This prospective, observational, nonrandomized study was performed at the Quinze-Vingts National Ophthalmology Hospital in Paris, France, on consecutive patients with nonprogressive duck keratoconus phenotypes undergoing implantation with AJL PRO + ICRS between August 2020 and March 2022. All patients completed follow-up visits at one and six months. The study was performed in accordance with the Declaration of Helsinki, with the approval of the Ethics Committee of the French Society of Ophthalmology (Institutional Review Board 00008855). The inclusion criteria were the presence of type 2 keratoconus (“*Duck* phenotype”) according to the Fernandez-Vega/Alfonso morphological keratoconus classification, defined as a paracentral topographic phenotype with noncoincident topographic and coma axes, associated with contact lens intolerance, clear central cornea, a minimal corneal stromal thickness of 350 µm in the proposed implantation area, patient age ≥ 18 years and postoperative follow-up for at least six months. The keratoconus diagnosis and the determination of the keratoconus profile were facilitated by the use of AS-OCT combined with a Placido disc (MS-39, CSO, Firenze, Italy) [24]. The MS-39 system has been shown to have good repeatability for topographic parameters^16^ and for AS-OCT measurements in normal and keratoconic eyes [25]. The exclusion criteria were prior corneal or intraocular surgery, a history of herpetic keratitis, diagnosed autoimmune disease, systemic connective tissue disease, cataract, a history of glaucoma or retinal detachment, macular degeneration or other retinopathies, neuro-ophthalmic disease or a history of ocular inflammation. 

### 2.2. Examination Protocol

All patients underwent a complete ophthalmologic examination before ICRS surgery and one and six months after surgery. This examination included determinations of uncorrected distance visual acuity (UDVA) and corrected distance visual acuity (CDVA) on manifest refraction. A Scheimpflug camera with a Placido disc was used to obtain simulated corneal topography, asphericity and corneal aberrometry data. An AS-OCT pachymetry map (16 mm scan diameter, 25 radials, 1024 axial scans) was used to provide an 8 mm diameter scan of total corneal thickness and epithelial thickness mapping data (the central 3 mm zone and 24 peripheral sites in the 3 mm to 8 mm zones centered on the pupillary axis).

### 2.3. Surgical Technique 

All procedures were performed under topical anesthesia. The center of the pupil was marked, and a disposable suction ring was centered on the pupil. A channel was subsequently created with a femtosecond laser (Wavelight FS 200, Alcon Laboratories, Inc., Fortworth, TX, USA) at 75% of the thickness of the cornea. The incision was performed on the steepest keratometry axis. The laser software was programmed according to the ICRS type, with a 5 mm diameter ring requiring an inner diameter of 4.4 mm and an outer diameter of 5.7 mm. The channel and incision were created with an energy of 1.20 mJ. ICRSs were implanted with dedicated forceps under fully aseptic conditions. The segments were set in their final positions with the aid of a Sinskey hook. Postoperative treatment consisted of combined antibiotic (tobramycin, 3 mg/mL) and steroid (dexamethasone, 1 mg/mL) eye drops (Tobradex; Alcon Laboratories Inc., Fort Worth, TX, USA) administered three times daily for two weeks and eye lubricant (Vismed, Horus Pharma, France) for one month after surgery.

### 2.4. Intrastromal Corneal Ring Segments 

**Figure 1:**
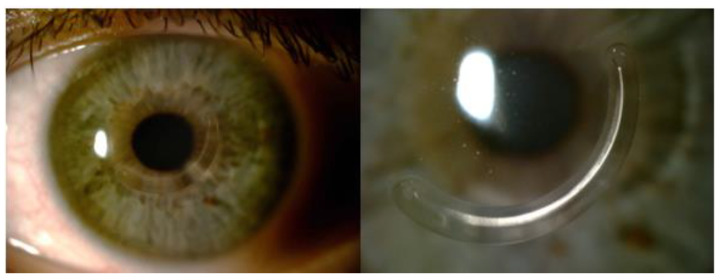
Slit-lamp photography 6 months after AJL PRO + implantation. Base width gradually increases from 800 to 1200 μm and thickness increases from 150 to 250 μm, with a diameter of 5 mm and arc length of 160 degrees.

AJL PRO + intrastromal ring segments consist of polymethylmethacrylate (PMMA) and have a triangular cross-section with a flat base. The base width and thickness may increase in a clockwise or counterclockwise manner. AJL PRO + ring segments with a diameter of 5 mm and an arc length of 160° were implanted. The base width and thickness gradually increased from 600 to 800 μm and from 0.15 to 0.25 mm (type A) or 0.15 to 0.30 mm (type B), respectively (Figure 1). The appropriate thickness and position were selected by the surgeon based on keratoconus grade, topographic astigmatism and manifest refraction. The midpoint of each ICRS segment was aligned on the flat topographical axis. The ring was inserted with the widest and thickest end of the segment oriented toward the steepest hemi-meridian.

### 2.5. Differential Map

Sagittal map keratometry and epithelial thickness changes were displayed on differential maps by the MS39 system. The mean difference was calculated in the central 3 mm, 3–5 mm and 5–8 mm of the cornea and plotted in a figure. The data for the left eyes were transformed by symmetry with respect to the vertical axis to obtain directly comparable data for both eyes. 

**Figure 2:**
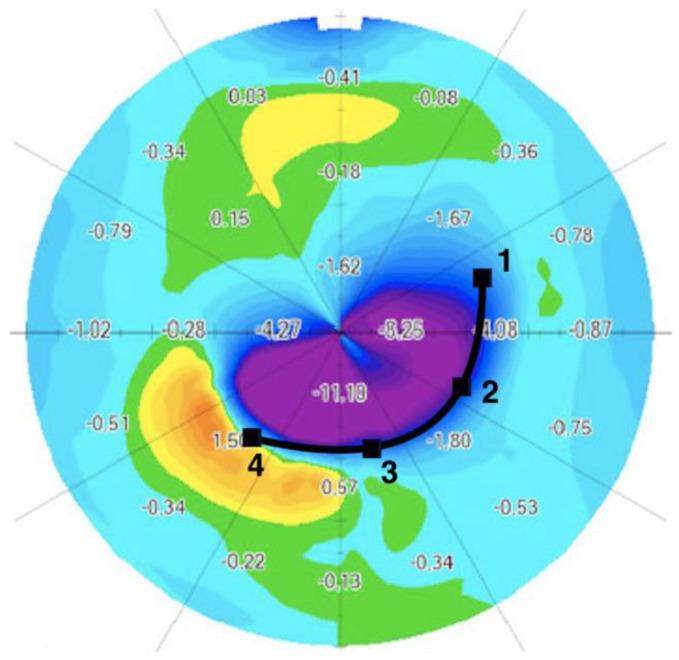
Keratometry variation after AJL PRO + ICRS implantation to treat duck-type keratoconus. The ICRS is split into sections defined by 4 equidistant points, from the thinnest part (point 1) to the widest (point 4). For each point, we calculated the change in anterior sagittal keratometry in diopters.

With the help of corneal thickness differential maps, on which the location of the ICRS in the cornea is well visualized, we divided the asymmetric ICRS according to four equidistant points, from the thinnest and narrowest end (point 1: 150 µm thick and 600 µm wide) to the thickest and widest end (point 4: 250 or 300 µm thick and 800 µm wide). For each point, we calculated the anterior sagittal keratometry change, in diopters (Figure 2).

The MS39 system locates the apex of the cone. The pachymetry and epithelial thickness at the apex of the cone and its distance from the center of the cornea were measured before and six months after ICRS implantation.

### 2.6. Corneal Aberrations

Corneal aberrations were measured with the MS39 system. Zernike coefficients and root-mean-square (RMS) values were calculated for a pupil diameter of 5 mm. We analyzed anterior, posterior and total corneal aberrations for the following RMS groups: high-order aberrations (HOAs), low-order aberrations (LOAs), coma (and coma axis), trefoil, spherical aberration and higher-order astigmatism aberrations.

### 2.7. Data Analysis

The efficacy of ICRS implantation for correcting astigmatism was evaluated by the Alpins method, a vectorial astigmatism outcome analysis based on three fundamental vectors, as follows: the target-induced astigmatism (TIA) vector, surgically induced astigmatism (SIA) vector and difference vector, as described by Alpins [26,27]. We determined the correction index (ratio of the SIA to the TIA), the flattening index (ratio of the flattening effect–SIA multiplied by the cosine of twice the angle of the error to the TIA) and the refractive astigmatism angle of error.

### 2.8. Safety and Efficacy 

The efficacy index was calculated by dividing the mean postoperative UDVA by the mean preoperative CDVA. The safety index was assessed by dividing the mean postoperative CDVA by the mean preoperative CDVA. 

### 2.9. Statistical Analysis

Results are presented as means ± standard deviations (SDs) for continuous variables and as proportions (%) for categorical variables. Snellen visual acuities were converted into logarithms of the minimum angle of resolution (logMAR) units for analysis. D’Agostino–Pearson tests were used to assess the normality of the data distribution, and nonparametric statistic tests were applied. For paired tests, 25 eyes were required to detect differences of 0.10 logMAR in visual acuity measurements between consecutive visits, assuming a statistical power of 85% and an alpha error of 0.05. The Wilcoxon matched-pairs test was used for statistical comparisons between preoperative and postoperative continuous data. The Kruskal–Wallis test was used to compare continuous data, as appropriate. For binary outcomes, the stratified Cochran chi-squared test was used for intergroup comparisons of proportions. Pearson’s correlation tests were used to explore the relationship between values. Corrected *p*-values less than 0.05 were considered statistically significant. Statistical analysis was performed with SPSS software for Windows (version 20.0; SPSS, Inc., Chicago, IL, USA).

## 3. Results

### 3.1. Study Population 

We included 33 keratoconus eyes from 29 consecutive patients, 15 women (52%) and 14 men (48%). The mean age at presentation was 32.4 ± 10.5 years. Demographic data and ocular characteristics at inclusion are summarized in Table 1. No intraoperative or postoperative complications occurred during the study. Type A ICRSs (0.15 to 0.25 mm) were implanted in 10 eyes, and type B ICRSs (0.15 to 0.30 mm) were implanted in 23 eyes, with an arc length of 160° (33 eyes), according to topographic and tomographic patterns.

### 3.2. Visual Acuity and Refraction 

**Figure 3:**
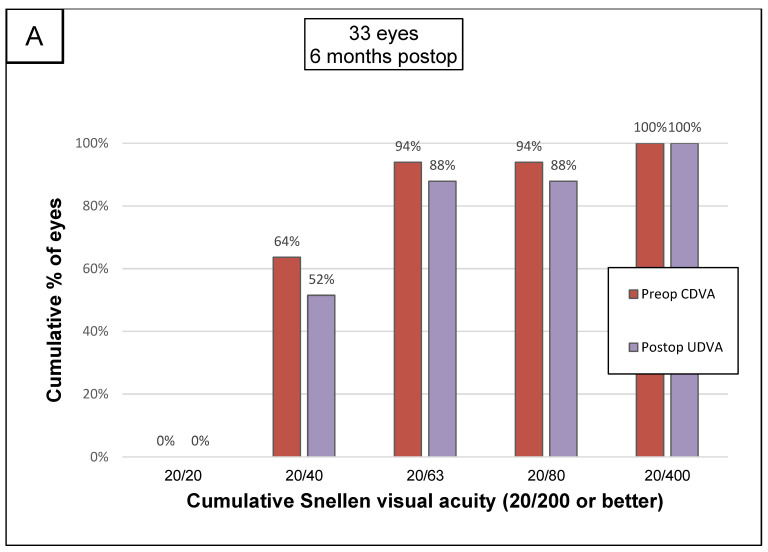

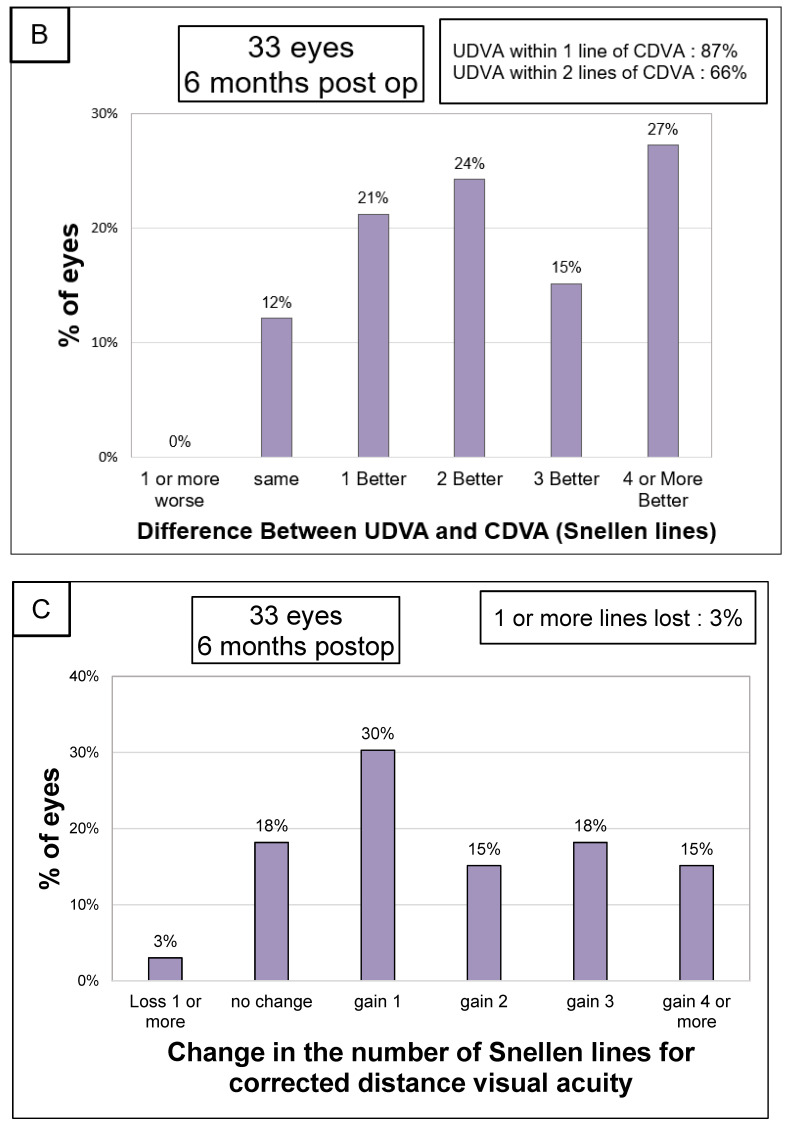

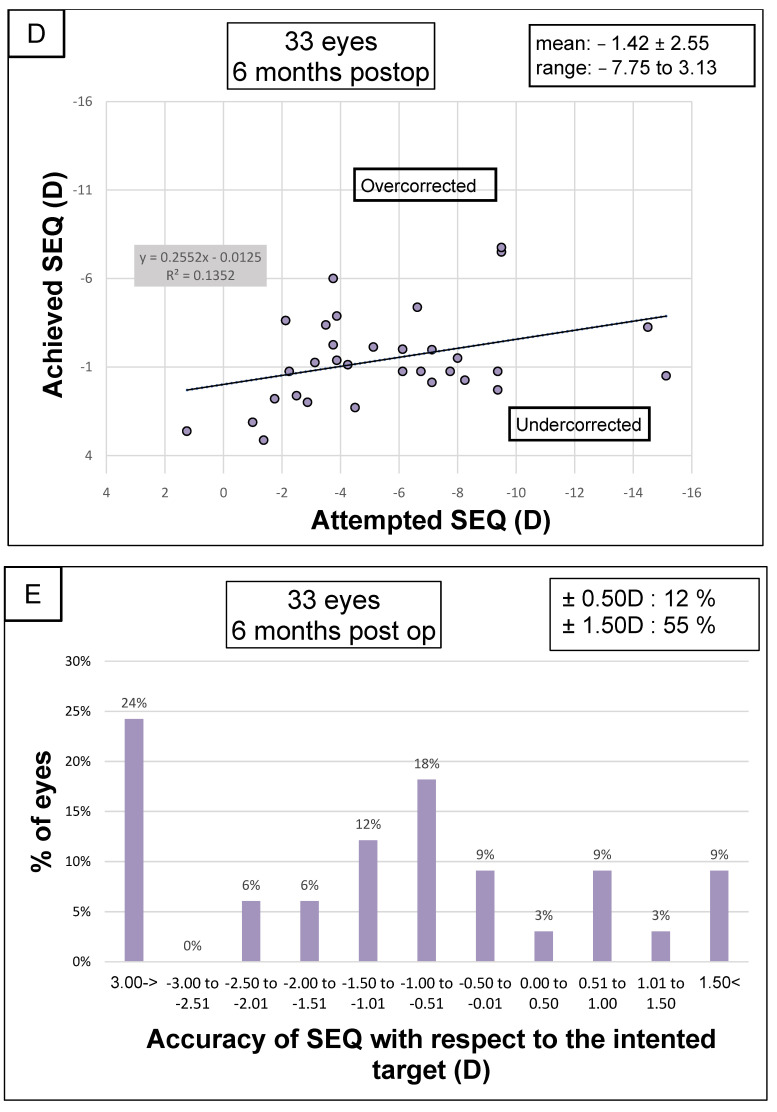

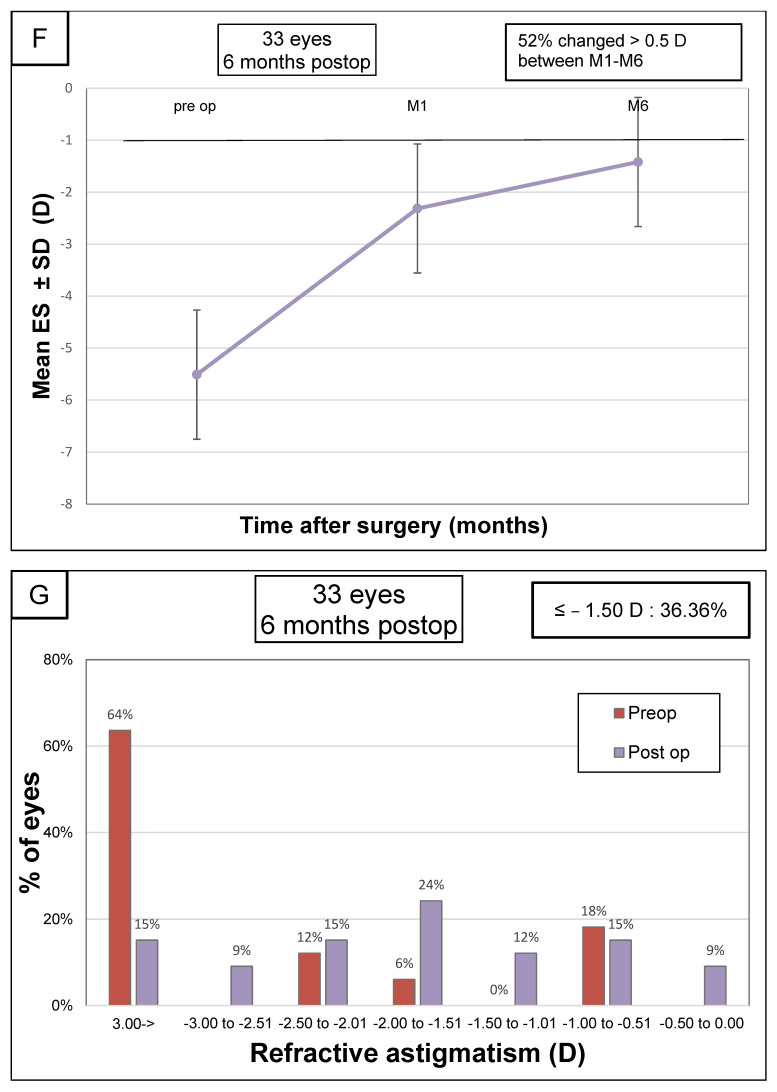

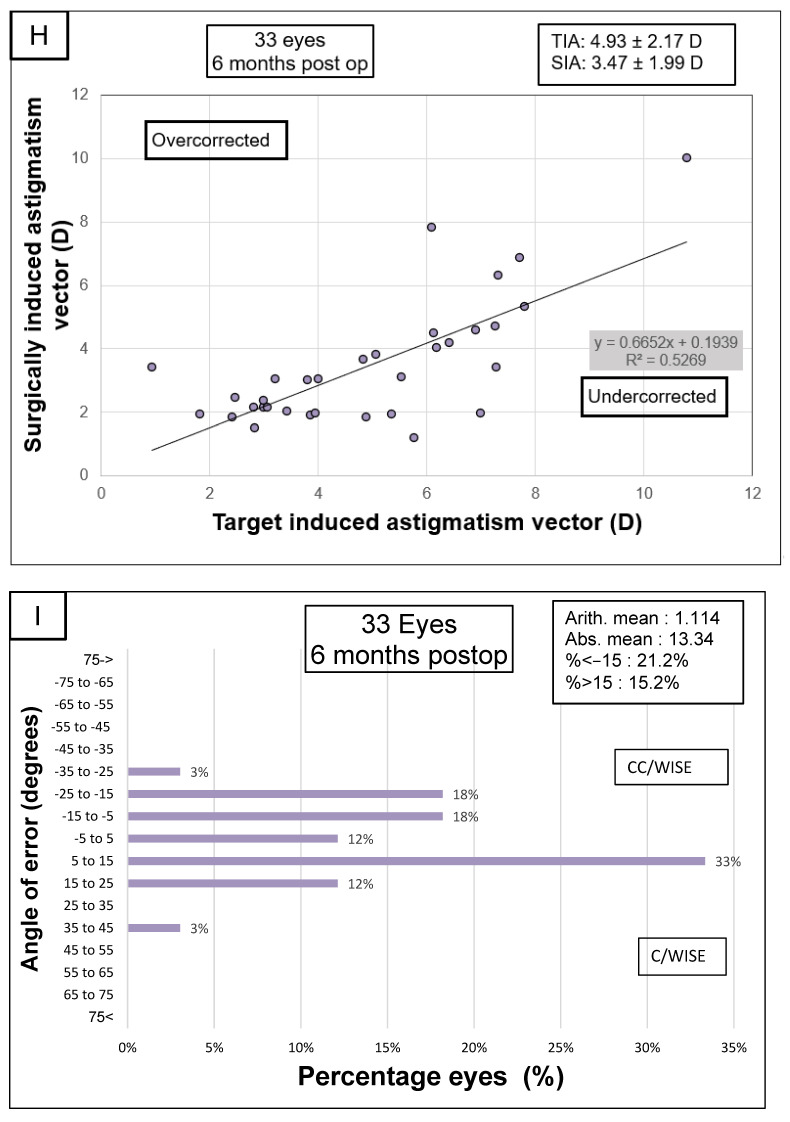
Standard graphs for corneal refractive surgery. (**A**): At 6 months after surgery, 52% of patients had a UDVA of 20/40 or better. (**B**): Difference between UDVA and CDVA 6 months after surgery (Snellen): 66% of patients had a postoperative UDVA within 2 lines of CDVA. (**C**): Change in the number of Snellen lines for CDVA between the preoperative examination and the examination six months after surgery: 3% (*n* = 1) lost 1 Snellen line, and 48% of patients gained 2 or more lines of CDVA. (**D**): SEQ targeted vs. achieved: the coefficients of determination are shown. (**E**): SEQ accuracy: for 55% of the eyes implanted, the SEQ was within ± 1.5 diopters D of the target value. (**F**): Stability of spherical equivalent refraction over the 6 months following surgery: a major part of the correction was obtained at 1 month, and the result was stable at 6 months. (**G**): Refractive astigmatism (**D**): preoperatively, 64% of patients had a refractive astigmatism of more than 3 D. Postoperatively, 36.36% of patients had a refractive astigmatism of less than 1.50 D. (**H**): Target-induced astigmatism (TIA) vs. surgically induced astigmatism (SIA): the mean magnitude of SIA was higher than that of TIA, indicating an overcorrection of corneal astigmatism. (**I**): Refractive astigmatism angle of error: the angle of error was accurate (between −15° and +15°) for 63.6% of eyes. UDVA: uncorrected distance visual acuity; CDVA: corrected distance visual acuity; SEQ: spherical equivalent; Pre: preoperative; 1: one month after surgery; 3: 3 months after surgery; 6: 6 months after surgery; D: diopters; TIA: target-induced astigmatism; SIA: surgically induced astigmatism; Arith. Mean: arithmetic mean angle of error; Abs. Mean: absolute mean angle of error; C: clockwise; CC: counterclockwise.

The principal visual results and refractive parameters are summarized in Table 2 and Figure 3. Follow-up assessments after surgery showed a significant improvement in both UDVA and CDVA. Following AJL PRO + ICRS implantation, there was a statistically significant change in UDVA from 0.75 ± 0.38 to 0.37 ± 0.24 logMAR (*p* < 0.001). At six months, we found a significant decrease in the spherical equivalent from –5.51D ± 3.68 to −1.42D ± 2.55 and in the refractive cylinder from −3.73 D ± 2.16 to −1.99 D ± 1.10 (*p* < 0.001). Twenty-nine of the eyes receiving implants (87%) gained at least one line of visual acuity, and one eye (3%) lost one line of visual acuity. At the end of the follow-up, 13 eyes (55%) had achieved MRSEs between +1.50 and −1.50 D. The mean magnitude of SIA was higher than that of TIA. The angle of error was accurate between −15° and +15° in 21 eyes (63.6%). 

### 3.3. Differential Map Analysis 

**Figure 4:**
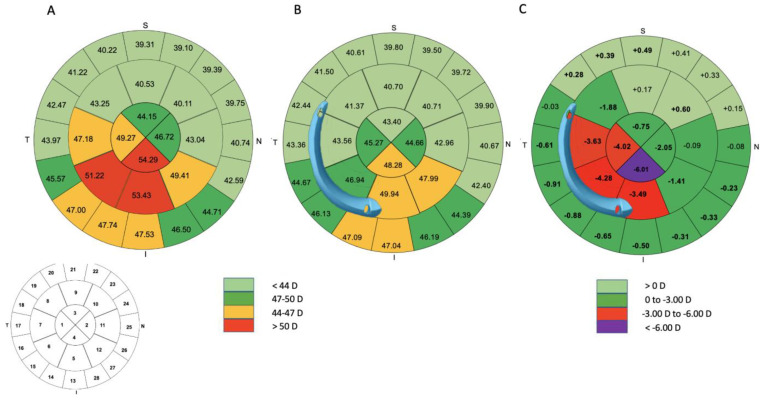
The mean sagittal anterior keratometry in 28 zones in the central 8 mm of the cornea is shown for the examinations performed before surgery (**A**) and 6 months after the implantation of one AJL PRO + ICRS (**B**). The average position of the ring is shown. The averaged differential map (**C**) shows that the flattening effect of the AS-ICRS is greater at the widest end than at the narrowest end. Significant results are shown in bold.

Table 3 and Figure 4 summarize the results for pre- and postoperative keratometry values for various areas in the central 8 mm of the cornea. A significantly greater flattening effect was obtained at the widest and thickest end of the segment, with a progressive decrease in flattening down to the narrowest and thinnest end (*p* < 0.001). At the thinnest and narrowest point in the ring (point 1), the mean keratometric correction induced was −2.94 D ± 1.84. At points 2 and 3, the mean correction was −5.14 D ± 2.26 and −7.37 D ± 3.38, respectively. Correction at the base of the AS ICRS, where the width and thickness were greatest (point 4), was −9.25 D ± 3.84 (Figure 2).

### 3.4. Safety and Efficacity

The six-month efficacy index (mean postoperative UDVA/mean preoperative CDVA) was 0.88, and the six-month safety index (ratio of postoperative to preoperative CDVA) for ICRS implantation was 1.45.

### 3.5. Topographic Results

Table 4 provides the corneal topographic outcomes. Significant flattening was observed on keratometry, with a decrease in mean keratometry values from 47.95 ± 3.94 to 44.95 ± 3.59 (*p* < 0.001) in the central 3 mm and from 46.98 ± 3.25 to 44.32 ± 2.89 (*p* < 0.001) in the central 5 mm. At the six-month follow-up examination, flat and steep keratometry (K1, K2) and corneal topographic astigmatism in the central 3 and 5 mm had decreased significantly in the anterior cornea (*p* <0.001). In addition, the anterior Q-factor in the central 8 mm improved from −0.96 ± 0.46 to −0.26 ± 0.60 (*p* < 0.001). The posterior Q-factor in the central 8 mm and 5 mm shifted to a more prolate profile, from −1.26 ± 0.56 to −1.67 ± 0.71 (*p* < 0.001) and from −1.30 ± 1.24 to −2.58 ± 2.83, respectively (*p* = 0.007). On posterior keratometry, we found a reduction in steep keratometry (K2) values, but no significant decrease in flat keratometry (K1) values (*p* = 0.15). The symmetry index front (SIF) decreased from 10.31 ± 3.14 to 6.42 ± 3.08 (*p* < 0.001) (Supplementary Table S1).

### 3.6. Pachymetric Analysis

**Figure 5:**
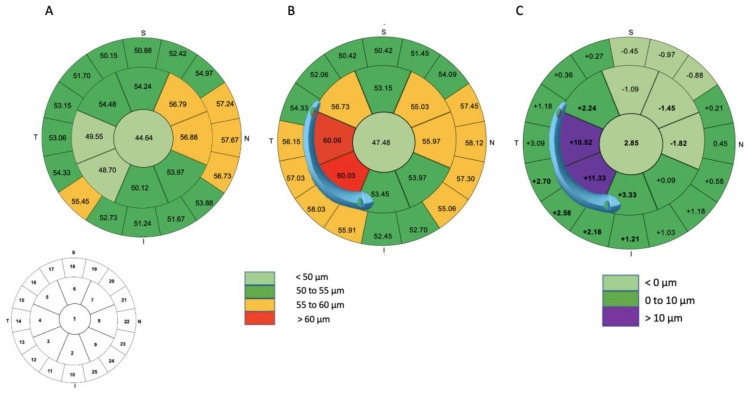
The mean epithelial thickness in 25 zones in the central 8 mm of the cornea is shown for the examinations performed before surgery (**A**) and 6 months after the implantation of one AJL PRO + ICRS (**B**). The mean difference in epithelial thickness (**C**) reveals a concentration of the epithelial remodeling effect on the inner part of the AS-ICRS, with stronger remodeling at the wider end than at the narrower end. Significant results are shown in bold.

Figure 5 and Table 5 summarize the results for pre- and postoperative epithelial thickness measurements in the central 8 mm of the cornea. A progressive thickening of the epithelium after ICRS implantation was noted. The differential epithelium thickness at the wider and thicker end was 11.33 ± 12.95 µm (*p* < 0.001), whereas that at the narrower and thinner end was 2.24 ± 5.67 µm (*p* = 0.01). A significant increase in the minimum corneal thickness from 437 ± 38.38 µm to 459 ± 42.09 µm was observed during the follow-up (*p* < 0.001). At the apex of the cone, the epithelial thickness increased from 44.91 ± 5.51 µm to 50.42 ± 7.06 µm (*p* = 0.002).

### 3.7. Aberrometry

Table 6 summarizes the pre- and postoperative corneal aberrometry data. With respect to total corneal HOAs, we observed a decrease in RMS in the central 5 mm of the cornea, from 1.93 ± 0.89 μm preoperatively to 1.69 ± 0.78 µm postoperatively (*p* = 0.02), with a marked reduction in RMS total coma aberration from 1.62 ± 0.81 µm to 0.99 ± 0.59 µm (*p* < 0.001). For anterior corneal aberrations, we found a decrease in RMS coma aberration from 2.12 ± 1.03 to 1.37 ± 0.85 (*p* < 0.001) and a decrease in RMS trefoil aberration from 0.87 ± 0.51 to 0.51 ± 0.38 (*p* < 0.001). No significant difference between preoperative and postoperative values was observed for posterior coma aberration (*p* = 0.06).

### 3.8. Correlation

Pearson’s correlation tests revealed the absence of a correlation between visual parameters, keratometric and aberrometric parameters and epithelial parameters.

## 4. Discussion

We found that the implantation of AJL PRO + ICRS improved refractive, keratometric and aberrometric readings and visual parameters in patients with duck-type keratoconus. Following implantation, an epithelial thickening of the central corneal area was observed, with a progressive thickening of the epithelium from the thinnest to the thickest part of the ICRS.

### 4.1. Clinical, Topographic and Aberrometric Parameters

AJL PRO + ICRS implantation led to a statistically significant change in UDVA from 0.24 ± 0.12 to 0.47 ± 0.21 for decimal-scale visual acuity (*p* < 0.001). UDVA improved in all patients due to a significant decrease in refractive parameters, such as astigmatism and the spherical equivalent, with a mean spherical equivalent myopic correction of 4.09 D (*p* < 0.001) after six months of follow-up.

Keratometry revealed a significant progressive flattening effect along the ICRS, with a greater average correction at the thickest and widest end. This led to an asymmetric decrease in topographic parameters, resulting in a significant decrease in SIF or SIB (Supplementary Table S1). Moreover, recent studies have reported an association between the loss of Snellen lines for CDVA and an increase in the I-S difference [28,29]. In typical keratoconus, coma aberration appears with a delayed wavefront in the inferior cornea and an advanced wavefront in the superior cornea, consistent with a vertical asymmetric corneal shape [30]. In this study, primary coma, which is known to be the principal clinically relevant high-order aberration in keratoconic corneas [31], improved significantly, by 38.8%, after AJL PRO + implantation. Many studies of symmetric or asymmetric ICRS treatment have reported a significant improvement in asymmetric aberrations, such as primary coma and coma-like aberrations, which are known to have a negative effect on vision quality [9,11,32,33].

Despite improvements in anterior corneal HOAs, there was no correlation between the improvement in anterior corneal HOAs and visual acuity six months after surgery. Moreover, we found significant differences in almost all posterior parameters. It has been suggested that the improvement in CDVA in keratoconic eyes is conditioned by the regularization of the posterior corneal surface of the cone, but we found no such correlation [34]. These findings suggest that a larger randomized cohort study should be performed to increase the power for the confirmation of these trends.

### 4.2. Pachymetric Analysis

Reinstein et al. described a doughnut-like epithelial thickness profile in keratoconus, with localized epithelial thinning in the center of the cone, surrounded by an annulus of thick epithelium [35]. They showed, by ultrasound imaging, that epithelial filling occurred in the concave anterior stromal groove produced after ICRS implantation for the treatment of myopia [22]. David et al. analyzed SD-OCT results for nonasymmetric ICRS implantation (Ferrara type) for keratoconus and found significant epithelial thickening in the internal zones juxtaposed with the ICRS; they showed that this epithelial thickening differed between ICRS and keratoconus types [23]. The stroma of the cone apex also seems to be remodeled following ICRS implantation, as also shown by the thickening of the epithelium^23^, and could be the result of stromal flattening. Our findings are consistent with these results, as we observed a significant epithelial thickening around the ring with a tendency to extend toward the apex, revealing an effect of the ICRS on the stroma.

AJL PRO + implantation induces an asymmetric flattening of the corneal surface due to the gradated shape of the implant. Stromal curvature variation reflects the progressive change in the stroma and partly dictates epithelial remodeling [36,37]. We observed progressive asymmetric epithelial thickening from the thinnest to the thickest part of the ICRS, with no smoothing of this epithelial profile at the six-month follow-up. These variations in the distribution of epithelial thickening can be accounted for by the compensatory function of epithelial remodeling, which reduces the underlying irregular redistribution of the stroma by increasing or decreasing the thickness of epithelial cell layers to “compensate” for variations along the length of the ICRS [38]. Epithelial thinning can decrease corneal curvature and HOAs [39]. We suggest that the asymmetric epithelial profile induced by the ICRS may help to decrease coma aberration after asymmetric ICRS implantation.

Additional procedures, such as crosslinking and transepithelial PRK, are more frequent after ICRS implantation for keratoconus management [16,18,40]. Transepithelial PRK typically involves the removal of a 50 μm layer of epithelium before the second step of PRK [19,21]. ICRS implantation induces an irregular epithelial profile, with an increase in epithelial thickness in certain areas, including, in particular, the inner part of the ICRS. A transepithelial procedure with the conventional 50 μm ablation depth might, therefore, result in incomplete treatment in the thickest areas. In this situation, mechanical debridement may be a useful option for ensuring the complete, safe removal of the epithelium before PRK. On the other hand, too much of the corneal stroma overlying the ICRS might be removed due to the overtreatment of these thin epithelial areas during PRK, leading to corneal stroma thinning and a possible increase in the risk of ICRS extrusion [41]. Given the epithelial mapping patterns that may be encountered after ICRS implantation, it would therefore appear sensible to analyze epithelial changes when combining this technique with other corneal procedures.

Finally, AJL PRO + ICRS implantation improves refractive, topographic and aberrometric outcomes, leading to a progressive epithelial thickening extending to the apex of the cornea, from the thinnest to the thickest part of the ICRS, at the six-month follow-up in cases of “duck-type” keratoconus. This asymmetric design helps to decrease anterior corneal coma and increases visual acuity.

## Figures and Tables

**Table 1:** Patient demographics and ocular characteristics at inclusion (*n* = 33).

Parameters	
Age (years)	32.42 ± 10.53 [18 to 60]
Sex	
Female	17
Male	16
Keratoconus type	
Duck	33
Amsler–Krumeich stage	
I	20
II	11
III	1
IV	1
UDVA (LogMAR)	0.75 ± 0.38 [0.10 to 1.40]
UDVA (Snellen)	20/100 [20/500 to 20/25]
CDVA (LogMAR)	0.30 ±0.18 [0.0 to 7.00]
CDVA (Snellen)	20/40 [20/100 to 20/25]
K mean at 3 mm (D)	47.95 ±3.94 [40.32 to 60.67]
Sphere (D)	−3.64 ± 3.36 [−11.75 to 2.25]
Cylinder (D)	−3.73 ± 2.16 [−8.75 to −0.75]
Spherical equivalent (D)	−5.51 ± 3.68 [−15.13 to 1.25]
Minimum corneal thickness (μm)	437 ± 39.38 [366 to 570]
Symmetry index front	10.31 ± 3.14 [3.14 to 15.69]

Values are presented as the mean ± standard deviation and [range]. UDVA: uncorrected distance visual acuity; CDVA: corrected distance visual acuity; K mean at 3 mm: mean keratometry value in the central 3 mm of the cornea; D = diopters.

**Table 2:** Vision and refractive results (*n* = 33).

Parameters	Preoperative	M6	Δ	*p*-Value *
UDVA (LogMAR)	0.75 ± 0.38 [0.10 to 1.40]	0.37 ± 0.24 [0.00 to 1.00]	−0.38 ± 0.29 [−1.10 to 0.00]	**<0.001**
UDVA (Snellen)	20/100 [20/500 to 20/25]	20/50 [20/200 to 20/20]		
CDVA (LogMar)	0.32 ± 0.19 [0.0 to 7.00]	0.12 ± 0.12 [0.10 to 0.40]	−0.20 ± 0.19 [−0.40 to 0.10]	**<0.001**
CDVA (Snellen)	20/40 [20/100 to 20/20]	20/25 [20/40 to 20/20]		
Sphere (D)	−3.64 ± 3.36 [−11.75 to 2.25]	−0.87 ± 2.63 [−7.00 to 4.75]	2.77 ± 3.01 [−3.50 to 11.50]	**<0.001**
Refractive cylinder (D)	−3.73 ± 2.16 [−8.75 to −0.75]	−1.99 ± 1.10 [−5.00 to −0.25]	1.74 ± 2.23 [−4.00 to 6.25]	**<0.001**
Spherical equivalent (D)	−5.51 ± 3.68 [−15.13 to 1.25]	−1.42 ± 2.55 [−7.75 to 3.13]	4.09 ± 3.62 [−2.25 to 14.63]	**<0.001**

* Wilcoxon signed-rank test. Values are presented as the mean ± standard deviation and [range]. Significant results are indicated in bold. M6: 6 months after surgery; Δ: difference between 6 months after surgery and preoperative values; UDVA: uncorrected distance visual acuity; CDVA: corrected distance visual acuity; D = diopters.

**Table 3:** Sagittal anterior keratometry in the central 8 mm (*n* = 33).

	Preoperative	M6	Δ	*p*-Value *
Central 3 mm				
Zone 1 (D)	49.29 ± 4.43 [42.41 to 63.70]	45.27 ± 3.50 [39.50 to 54.27]	−4.02 ± 3.03 [−9.68 to 1.58]	**<0.001**
Zone 2 (D)	46.72 ± 4.35 [38.22 to 57.64]	44.66 ± 3.44 [38.97 to 54.01]	−2.05 ± 2.73 [−8.41 to 4.10]	**<0.001**
Zone 3 (D)	44.15 ± 5.18 [32.57 to 56.10]	43.40 ± 4.55 [32.76 to 53.76]	−0.75 ± 1.83 [−6.24 to 4.59]	**0.01**
Zone 4 (D)	54.29 ± 5.84 [45.44 to 70.83]	48.28 ± 7.24 [36.99 to 69.07]	−6.01 ± 4.85 [−13.70 to 5.64]	**<0.001**
Central 3 to 5 mm				
Zone 5 (D)	53.43 ± 3.46 [45.97 to 64.06]	49.94 ± 3.72 [38.39 to 55.70]	−3.49 ± 4.02 [−17.65 to 1.74]	**<0.001**
Zone 6 (D)	51.22 ± 3.81 [43.88 to 63.73]	46.94 ± 3.45 [38.73 to 53.46]	−4.28 ± 2.57 [−10.27 to −1.00]	**<0.001**
Zone 7 (D)	47.18 ± 4.05 [40.80 to 58.43]	43.56 ± 3.13 [38.31 to 50.00]	−3.63 ± 2.58 [−12.71 to 0.30]	**<0.001**
Zone 8 (D)	43.25 ± 3.46 [36.53 to 50.93]	41.37 ± 3.11 [35.18 to 47.63]	−1.88 ± 1.38 [−4.93 to 0.92]	**<0.001**
Zone 9 (D)	40.53 ± 2.92 [33.79 to 46.73]	40.70 ± 3.06 [33.92 to 46.56]	0.17 ± 0.87 [−1.92 to 2.62]	0.13
Zone 10 (D)	40.11 ± 2.73 [32.80 to 45.88]	40.71 ± 2.85 [33.96 to 46.25]	0.60 ± 0.62 [−0.54 to 2.04]	**<0.001**
Zone 11 (D)	43.04 ± 2.64 [36.28 to 47.20]	42.96 ± 2.55 [36.98 to 46.82]	−0.09 ± 0.86 [−1.91 to 1.29]	0.28
Zone 12 (D)	49.41 ± 2.94 [43.95 to 57.30]	47.99 ± 3.15 [39.03 to 53.58]	−1.41 ± 2.66 [−10.32 to 2.50]	**0.002**
Central 5 to 8 mm				
Zone 13 (D)	47.53 ± 2.49 [42.77 to 53.41]	47.04 ± 2.52 [41.70 to 52.72]	−0.50 ± 0.75 [−3.20 to 0.97]	**<0.001**
Zone 14 (D)	47.74 ± 2.56 [43.12 to 53.44]	47.09 ± 2.57 [42.07 to 52.26]	−0.65 ± 0.82 [−3.94 to 0.82]	**<0.001**
Zone 15 (D)	47.00 ± 2.65 [43.47 to 53.54]	46.13 ± 2.65 [41.60 to 52.16]	−0.88 ± 0.79 [−3.83 to 0.01]	**<0.001**
Zone 16 (D)	45.57 ± 2.80 [40.84 to 53.31]	44.67 ± 2.77 [40.47 to 51.48]	−0.91 ± 0.73 [−3.17 to 0.54]	**<0.001**
Zone 17 (D)	43.97 ± 2.87 [38.64 to 52.06]	43.36 ± 2.87 [38.86 to 50.71]	−0.61 ± 0.86 [−2.33 to 1.90]	**<0.001**
Zone 18 (D)	42.47 ± 2.79 [37.44 to 49.51]	42.44 ± 2.99 [37.21 to 50.77]	−0.03 ± 0.81 [−1.33 to 1.29]	0.42
Zone 19 (D)	41.22 ± 2.70 [36.85 to 47.24]	41.50 ± 2.75 [36.17 to 48.21]	0.28 ± 0.67 [−0.98 to 1.34]	0.01
Zone 20 (D)	40.22 ± 2.58 [35.60 to 46.30]	40.61 ± 2.58 [35.44 to 45.60]	0.39 ± 0.72 [−0.92 to 2.41]	**0.002**
Zone 21 (D)	39.31 ± 2.74 [33.97 to 45.86]	39.80 ± 2.75 [34.70 to 45.30]	0.49 ± 1.01 [−2.65 to 2.16]	**0.004**
Zone 22 (D)	39.10 ± 2.58 [34.09 to 45.89]	39.50 ± 2.88 [32.00 to 45.39]	0.41 ± 1.16 [−4.00 to 2.85]	0.03
Zone 23 (D)	39.39 ± 2.58 [34.04 to 46.02]	39.72 ± 2.84 [32.77 to 45.67]	0.33 ± 1.11 [−4.53 to 2.48]	0.05
Zone 24 (D)	39.75 ± 2.53 [34.69 to 45.89]	39.90 ± 2.49 [34.79 to 45.46]	0.15 ± 0.60 [−1.30 to 1.21]	0.07
Zone 25 (D)	40.74 ± 2.29 [35.88 to 45.96]	40.67 ± 2.30 [35.98 to 45.45]	−0.08 ± 0.62 [−1.53 to 1.08]	0.26
Zone 26 (D)	42.59 ± 2.26 [37.97 to 46.12]	42.40 ± 2.22 [37.39 to 46.16]	−0.23 ± 0.58 [−1.77 to 1.10]	**0.02**
Zone 27 (D)	44.71 ± 2.26 [40.72 to 49.01]	44.39 ± 2.66 [37.97 to 49.30]	−0.33 ± 0.99 [−4.81 to 1.26]	**0.03**
Zone 28 (D)	46.50 ± 2.41 [42.75 to 51.53]	46.19 ± 2.54 [40.95 to 51.19]	−0.31 ± 0.88 [−2.19 to 1.99]	**0.02**

* Wilcoxon signed-rank test. All values are presented as the mean ± standard deviation and [range]. Significant results are indicated in bold.

**Table 4:** Keratometry and asphericity results (*n* = 33).

	Preoperative	M6	Δ	*p*-Value *
Anterior surface				
K1 (D)	45.63 ± 3.73 [38.58 to 57.78]	43.61 ± 3.52 [37.88 to 52.90]	−2.02 ± 2.17 [−5.97 to 1.57]	**<0.001**
K2 (D)	50.57 ± 4.48 [42.10 to 63.86]	46.38 ± 3.89 [40.53 to 57.05]	−4.18 ± 2.01 [−8.15 to −0.79]	**<0.001**
Km 3 mm (D)	47.95 ± 3.94 [40.32 to 60.67]	44.95 ± 3.59 [39.32 to 54.90]	−3.00 ± 1.97 [−6.42 to 0.22]	**<0.001**
Km 5 mm (D)	46.98 ± 3.25 [40.09 to 57.14]	44.32 ± 2.89 [39.80 to 51.97]	−2.66 ± 1.49 [−5.17 to 0.02]	**<0.001**
Astigmatism 3 mm (D)	4.93 ± 2.17 [0.93 to 10.79]	2.74 ± 1.63 [0.98 to 8.21]	−2.19 ± 1.57 [−5.17 to 1.89]	**<0.001**
Astigmatism 5 mm (D)	3.64 ± 1.62 [1.17 to 7.53]	2.22 ± 1.46 [0.45 to 7.32]	−1.43 ± 1.04 [−3.98 to 0.60]	**<0.001**
Q-factor (8 mm)	−0.96 ± 0.46 [−1.78 to −0.18]	−0.26 ± 0.60 [−1.67 to 0.75]	0.67 ± 0.53 [−1.03 to 1.69]	**<0.001**
SIF 3 mm (D)	10.31 ± 3.14 [3.14 to 15.69]	6.42 ± 3.08 [0.71 to 12.94]	−3.89 ± 2.83 [−11.79 to 0.73]	**<0.001**
Posterior surface				
K1 (D)	−6.94 ± 0.78 [−9.42 to −5.85]	−6.88 ± 0.71 [ −8.89 to −5.90]	0.06 ± 0.32 [−0.48 to 0.83]	0.15
K2 (D)	−7.80 ± 0.90 [−10.47 to −6.45]	−7.39 ± 0.73 [−9.31 to −6.38]	0.40 ± 0.35 [−0.12 to 1.16]	**<0.001**
Km 3 mm (D)	−7.35 ± 0.80 [−9.91 to −6.16]	−7.14 ± 0.71 [−9.09 to −6.29]	0.21 ± 0.28 [−0.17 to 0.83]	**<0.001**
Km 5 mm (D)	−6.95 ± 0.61 [−8.85 to −6.07]	−6.77 ± 0.54 [−8.24 to −6.03]	0.19 ± 0.18 [−0.09 to 0.61]	**<0.001**
Astigmatism 3 mm (D)	−0.85 ± 0.39 [−2.32 to −0.16]	−0.53 ± 0.30 [−1.35 to −0.07]	0.32 ± 0.32 [−0.69 to 0.97]	**<0.001**
Astigmatism 5 mm (D)	−0.61 ± 0.28 [−1.59 to −0.12]	−0.39 ± 0.20 [−0.90 to −0.08]	0.22 ± 0.21 [−0.17 to 0.69]	**<0.001**
Q-factor (8 mm)	−1.26 ± 0.56 [−2.30 to −0.17]	−1.67 ± 0.71 [−3.88 to −0.56]	−0.41 ± 0.41 [−1.86 to 0.38]	**<0.001**
Q-factor (5 mm)	−1.30 ± 1.24 [−3.65 to 2.52]	−2,58 ± 2.83 [−7.72 to 3.72]	−1.28 ± 2.86 [−6.73 to 3.53]	**0.007**

* Wilcoxon signed-rank test. All values are presented as the mean ± standard deviation and [range]. Significant results are indicated in bold. M6: 6 months after surgery; Δ: difference between values obtained six months after surgery and those obtained before surgery; K1: flat meridian; K2: steep meridian; Km: mean keratometry value; D = diopters; SIF: symmetry index front.

**Table 5:** Epithelial thickness in the central 8 mm (*n* = 33).

	Preoperative	M6	Δ	*p*-Value *
Central 3 mm				
Zone 1 (μm)	44.64 ± 4.26 [36.00 to 54.00]	47.48 ± 6.85 [37.00 to 64.00]	2.85 ± 5.93 [−8.00 to 18.00]	**0.004**
Central 3 to 5 mm				
Zone 2 (μm)	50.12 ± 4.88 [39.00 to 62.00]	53.45 ± 8.97 [39.00 to 83.00]	3.33 ± 9.19 [−8.00 to 36.00]	**0.02**
Zone 3 (μm)	48.70 ± 4.47 [38.00 to 56.00]	60.03 ± 11.95 [44.00 to 91.00]	11.33 ± 12.95 [−8.00 to 45.00]	**<0.001**
Zone 4 (μm)	49.55 ± 4.27 [39.00 to 59.00]	60.06 ± 11.00 [45.00 to 90.00]	10.52 ± 12.46 [−9.00 to 39.00]	**<0.001**
Zone 5 (μm)	54.48 ± 4.89 [41.00 to 64.00]	56.73 ± 7.40 [40.00 to 73.00]	2.24 ± 5.67 [−11.00 to 15.00]	**0.01**
Zone 6 (μm)	54.24 ± 5.20 [46.00 to 70.00]	53.15 ± 4.52 [46.00 to 63.00]	−1.09 ± 4.96 [−11.00 to 12.00]	0.09
Zone 7 (μm)	56.48 ± 4.80 [48.00 to 71.00]	55.03 ± 5.82 [47.00 to 72.00]	−1.45 ± 4.84 [−12.00 to 15.00]	**0.04**
Zone 8 (μm)	56.79 ± 5.57 [50.00 to 74.00]	55.97 ± 5.85 [47.00 to 74.00]	−1.82 ± 4.33 [−11.00 to 6.00]	**0.01**
Zone 9 (μm)	53.88 ± 4.14 [46.00 to 63.00]	53.97 ± 8.33 [38.00 to 82.00]	0.09 ± 6.24 [−8.00 to 22.00]	0.47
Central 5 to 8 mm				
Zone 10 (μm)	51.24 ± 5.26 [40.00 to 61.00]	52.45 ± 6.21 [42.00 to 66.00]	1.21 ± 5.73 [−6.00 to 20.00]	**0.11**
Zone 11 (μm)	52.73 ± 5.75 [39.00 to 64.00]	55.91 ± 6.26 [43.00 to 73.00]	2.18 ± 6.28 [−7.00 to 25.00]	**0.03**
Zone 12 (μm)	55.45 ± 5.36 [43.00 to 68.00]	58.03 ± 5.37 [48.00 to 75.00]	2.58 ± 5.63 [−9.00 to 23.00]	**0.006**
Zone 13 (μm)	54.33 ± 6.09 [37.00 to 65.00]	57.03 ± 5.06 [46.00 to 65.00]	2.70 ± 6.41 [−7.00 to 27.00]	**0.01**
Zone 14 (μm)	53.06 ± 5.34 [36.00 to 62.00]	56.15 ± 5.12 [44.00 to 67.00]	3.09 ± 6.83 [−7.00 to 29.00]	**0.007**
Zone 15 (μm)	53.15 ± 4.26 [42.00 to 63.00]	54.33 ± 5.32 [46.00 to 69.00]	1.18 ± 5.68 [−7.00 to 21.00]	0.12
Zone 16 (μm)	51.70 ± 4.77 [35.00 to 62.00]	52.06 ± 4.23 [46.00 to 62.00]	0.36 ± 4.76 [−9.00 to 13.00]	0.33
Zone 17 (μm)	50.15 ± 3.89 [42.00 to 59.00]	50.42 ± 3.30 [44.00 to 56.00]	0.27 ± 3.49 [−7.00 to 8.00]	0.33
Zone 18 (μm)	50.88 ± 4.22 [44.00 to 58.00]	50.42 ± 3.31 [42.00 to 55.00]	−0.45 ± 3.80 [−10.00 to 5.00]	0.25
Zone 19 (μm)	52.42 ± 3.60 [46.00 to 59.00]	51.45 ± 3.00 [47.00 to 59.00]	−0.97 ± 3.39 [−8.00 to 5.00]	0.05
Zone 20 (μm)	54.97 ± 3.39 [49.00 to 61.00]	54.09 ± 3.34 [47.00 to 62.00]	−0.88 ± 3.40 [−8.00 to 7.00]	0.07
Zone 21 (μm)	57.24 ± 3.59 [47.00 to 66.00]	57.45 ± 4.23 [50.00 to 70.00]	0.21 ± 3.67 [−9.00 to 10.00]	0.37
Zone 22 (μm)	57.67 ± 4.50 [48.00 to 67.00]	58.12 ± 4.51 [49.00 to 67.00]	0.45 ± 4.67 [−7.00 to 15.00]	0.29
Zone 23 (μm)	56.73 ± 4.10 [46.00 to 65.00]	57.30 ± 5.01 [45.00 to 67.00]	0.58 ± 4.17 [−11.00 to 8.00]	0.22
Zone 24 (μm)	53.88 ± 4.94 [42.00 to 63.00]	55.06 ± 5.71 [44.00 to 66.00]	1.18 ± 4.67 [−5.00 to 15.00]	0.08
Zone 25 (μm)	51.67 ± 4.79 [41.00 to 61.00]	52.70 ± 6.57 [40.00 to 65.00]	1.03 ± 4.93 [−7.00 to 16.00]	0.12

* Wilcoxon signed-rank test. All values are presented as the mean ± standard deviation and [range]. Significant results are indicated in bold.

**Table 6:** Corneal aberrometry results (*n* = 33).

	Preoperative	M6	Δ	*p*-Value *
Total cornea				
RMS Total Aberrations (μm)	2.84 ± 1.22 [0.87 to 4.68]	2.33 ± 0.99 [0.44 to 4.89]	−0.51 ± 0.68 [−2.34 to 0.94]	**<0.001**
RMS Total HOA (μm)	1.93 ± 0.89 [0.43 to 3.65]	1.69 ± 0.78 [0.32 to 3.53]	−0.24 ± 0.69 [−2.25 to 1.48]	**0.02**
RMS Spherical Aberration (μm)	−0.10 ± 0.21 [−0.49 to 0.48]	−0.03 ± 0.57 [−1.02 to 1.54]	0.07 ± 0.50 [−1.00 to 1.39]	0.20
RMS Coma (μm)	1.62 ± 0.81 [0.37 to 3.30]	0.99 ± 0.59 [0.23 to 2.42]	−0.63 ± 0.74 [−2.50 to 0.67]	**<0.001**
RMS Astigmatism (μm)	1.99 ± 1.03 [0.66 to 4.14]	1.48 ± 0.87 [0.12 to 4.36]	−0.51 ± 0.61 [−1.68 to 0.81]	**<0.001**
RMS Trefoil (μm)	0.73 ± 0.43 [0.08 to 1.71]	0.56 ± 0.40 [0.06 to 1.61]	−0.17 ± 0.51 [−1.25 to 0.66]	**0.03**
Anterior cornea				
RMS Spherical Aberration (μm)	−0.10 ± 0.26 [−0.57 to 0.65]	0.00 ± 0.43 [−0.83 to 1.29]	0.10 ± 0.34 [−0.80 to 1.01]	0.05
RMS Coma (μm)	2.12 ± 1.03 [0.43 to 4.14]	1.37 ± 0.85 [0.15 to 3.00]	−0.75 ± 0.73 [−2.35 to 0.77]	**<0.001**
Coma Axis (°)	88.88 ± 18.47 [57.00 to 151.00]	92.12 ± 25.74 [51.00 to 171.00]	−3.24 ± 26.48 [−58.00 to 96.00]	0.24
RMS Astigmatism (μm)	2.46 ± 1.21 [0.86 to 5.20]	1.61 ± 1.02 [0.36 to 5.32]	−0.86 ± 0.71 [−2.22 to 0.40]	**<0.001**
RMS Trefoil (μm)	0.87 ± 0.51 [0.10 to 1.91]	0.51 ± 0.38 [0.09 to 1.70]	−0.36 ± 0.55 [−1.30 to 0.58]	**<0.001**
Posterior cornea				
RMS Spherical Aberration (μm)	0.00 ± 0.09 [−0.21 to 0.23]	−0.07 ± 0.24 [−0.56 to 0.27]	−0.07 ± 0.27 [−0.60 to 0.37]	0.07
RMS Coma (μm)	0.50 ± 0.24 [0.08 to 1.07]	0.59 ± 0.39 [0.02 to 1.54]	0.08 ± 0.29 [−0.45 to 0.73]	0.06
Coma Axis (°)	267.73 ± 17.79 [228.00 to 308.00]	267.55 ± 36.10 [223.00 to 358.00]	0.08 ± 25.62 [−79.00 to 32.00]	0.48
RMS Astigmatism (μm)	0.51 ± 0.26 [0.15 to 1.45]	0.36 ± 0.23 [0.05 to 1.12]	−0.15 ± 0.29 [−0.82 to 0.33]	**0.003**
RMS Trefoil (μm)	0.16 ± 0.10 [0.02 to 0.51]	0.25 ± 0.14 [0.02 to 0.51]	0.09 ± 0.13 [−0.17 to 0.39]	**<0.001**

* Wilcoxon signed-rank test. All values are presented as the mean ± standard deviation and [range]. Significant results are indicated in bold. All values are calculated for a pupil diameter of 5 mm. M6: 6 months after surgery; Δ: difference between the values obtained before and six months after surgery; RMS: root mean square; HOA: higher-order aberrations.

## Data Availability

Data are available upon reasonable request.

## Supplementary Materials

The following supporting information can be downloaded at: https://www.mdpi.com/article/10.3390/jcm12041673/s1, Table S1: Keratoconus screening indices (n = 33).
