# Supplementary material for: Asymmetric Intrastromal Corneal Ring Segments with Progressive Base Width and Thickness for Keratoconus: Evaluation of Efficacy and Analysis of Epithelial Remodeling
Source: J Clin Med. 2023 Feb 20;12(4):1673. doi: 10.3390/jcm12041673 (PMC9962479; doi:10.3390/jcm12041673)
Supplement: Supplementary file 1 [file jcm-12-01673-s001.zip › jcm-2177839-supplementary.pdf]

Table S1. Keratoconus screening indices (n=33).

	Preoperative	M6	Δ	*P*-value*
		**Anterior surface**		
**SIF** **3 mm** (D)	10.31 ± 3.14 [3.14 to 15.69]	6.42 ± 3.08 [0.71 to 12.94]	-3.89 ± 2.83 [-11.79 to 0.73]	***<0.001***
**RMS/A** **6 mm** (μm/mm²)	0.37 ± 0.11 [0.11 to 0.60]	0.31 ± 0.09 [0.13 to 0.51]	-0.06 ± 0.09 [-0.26 to 0.17]	***<0.001***
**KvF** (μm)	45.36 ± 14.81 [12.60 to 80.60]	39.89 ± 12.46 [17.30 to 74.70]	-5.46 ± 9.69 [-28.90 to 8.60]	***<0.001***
**CSIF** (D)	3.62 ± 2.09 [-0.62 to 7.53]	0.81 ± 2.38 [-4.84 to 4.43]	-2.81 ± 2.06 [-7.61 to 1.06]	***<0.001***
**EIF** (μm)	45.33 ± 14.79 [12.63 to 80.56]	33.57 ± 14.53 [1.53 to 60.63]	-11.76 ± 19.13 [-75.99 to 8.59]	***<0.001***
**EIF Axis** (°)	259.30 ± 30.42 [201.00 to 319.00]	127.00 ± 29.49 [49.00 to 356.00]	-132.30 ± 25.44 [-74.00 to 52.00]	*0.17*
		**Posterior surface**		
**SIB** **3 mm** (D)	1.86 ± 0.68 [0.67 to 3.70]	-0.14 ± 0.89 [-2.09 to 1.55]	-2.00 ± 0.68 [-3.79 to -0.38]	***<0.001***
**RMS/A** **6 mm** (μm/mm²)	0.80 ± 0.27 [0.30 to 1.50]	1.07 ± 0.32 [0.71 to 2.05]	-0.26 ± 0.22 [-0.24 to 0.87]	***<0.001***
**KvB** (μm)	89.82 ± 25.52 [37 to 131.50]	73.36 ± 29.74 [27.10 to 136.70]	-16.46 ± 24.99 [-68.00 to 25.70]	***<0.001***
**CSIB** (D)	1.22 ± 0.81 [0.04 to 3.22]	1.90 ± 0.79 [0.03 to 3.69]	0.68 ± 0.52 [-1.15 to 1.59]	***<0.001***
**EIB** (μm)	89.82 ± 25.52 [36.95 to 131.48]	62.71 ± 38.94 [8.35 to 136.71]	-27.11 ± 28.80 [-99.81 to 21.83]	***<0.001***
**EIB Axis** (°)	260.79 ± 31.48 [215.00 to 318.00]	244.33 ± 71.27 [49.00 to 356.00]	-16.45 ± 69.22 [-269.00 to 100.00]	*0.09*

*Wilcoxon signed-rank test. All values are presented as the mean ± standard deviation and [range]. Significant results are indicated in bold. M6: 6 months after surgery; Δ: difference between values obtained six months after surgery and those obtained before surgery; D = diopters; SIF: symmetry index front; RMA/A = root mean square per unit of area; KvF = position and elevation of the highest point of ectasia on the anterior corneal surface; CSIF = center surrounding index of the anterior curvature ; EIF = ectatic index of the anterior surface; SIB: symmetry index back; KvB = position and elevation of the highest point of ectasia on the posterior corneal surface; CSIB = center surrounding index of the posterior curvature ; EIB = ectatic index of the posterior surface
